# Different Forms and Proportions of Exogenous Nitrogen Promote the Growth of Alfalfa by Increasing Soil Enzyme Activity

**DOI:** 10.3390/plants11081057

**Published:** 2022-04-13

**Authors:** Yi Zhao, Yuqiang Wang, Shengnan Sun, Wentao Liu, Ling Zhu, Xuebing Yan

**Affiliations:** 1 College of Animal Science and Technology, Yangzhou University, Yangzhou 225009, China; mx120200857@yzu.edu.cn (Y.Z.); wangyuqiang202066@163.com (Y.W.); 18852725916@163.com (W.L.); mx120210898@yzu.edu.cn (L.Z.); yxbbjzz@163.com (X.Y.); 2 Joint International Research Laboratory of Agriculture and Agri-Product Safety, The Ministry of Education of China, Yangzhou 225009, China

**Keywords:** amylase, nitrite reductase, ammonium nitrogen, nitrate nitrogen, nitrogen fertilization

## Abstract

Nitrogen fertilization is a simple and effective field management strategy for increasing plant productivity, but the regulatory mechanisms of nitrogen forms and proportions on soil nutrients and plant growth remain unclear. Therefore, we investigated soil enzyme activities and nutrient contents of alfalfa under different forms and proportions of exogenous nitrogen addition. Results showed that nitrogen input significantly increased the activity of three oxidoreductases (hydroxylamine reductase, nitrate reductase, and nitrite reductase) while having no significant effects on urease. A high proportion of ammonium nitrogen significantly increased neutral protease activity. The amylase activity markedly increased under mixed-nitrogen addition but decreased under single-nitrogen addition. Additionally, the contents of soil nutrients (soil organic matter, total nitrogen, nitrate nitrogen, ammonium nitrogen, available phosphorus, and available potassium) were significantly increased under different exogenous nitrogen inputs, which drove the changes in enzyme activities. Further, nitrogen addition also improved the biomass and nitrogen content of alfalfa. These findings indicated that applying different forms and proportions of exogenous nitrogen may stimulate soil enzyme activities, which will accelerate the transformation of nutrients and then promote alfalfa growth.

## 1. Introduction

Soil is a complex micro-environment; the exogenous nitrogen directly acts on the soil, thereby impacting the soil nutrient balance. For instance, nitrogen fertilization increases the nitrate nitrogen and ammonium nitrogen content of grassland soil [1], but it aggravates the degradation of organic carbon, which leads to nitrogen assimilation [2]. Soil enzymes are derived from the cell secretions of animals, plants, and microorganisms in the soil and the decomposition products of their residues. Enzymes accumulate in the soil in the form of free enzymes or absorb on the surface of soil particles [3]. Most enzymes that are frequently used to reflect soil carbon and nitrogen conversion can be divided into two groups: oxidoreductases and hydrolases. Soil oxidoreductases mainly include hydroxylamine reductase, nitrate and nitrite reductase, etc., which are directly involved in the denitrification process of soil nitrogen. Soil hydrolase (such as urease, amylase, and neutral protease) hydrolyzes protein and other macromolecular substances into simple and small molecules that are easy to be absorbed and utilized by plants. The activities of enzymes in soil which are known to be affected by nitrogen forms and the proportion of nitrogen are especially sensitive to the rate of nitrogen fertilization [4]. Nitrogen fertilization may have indirect effects on the activities of soil enzymes via changes in soil physicochemical properties, such as pH [5] and soil organic matter [6,7]. Meanwhile, increasing nitrogen availability affects the function and activity of soil enzymes to a certain extent through alleviating the nitrogen limitation to promote the decomposition of soil organic matter, as well as stimulate plant growth, thereby changing the soil carbon and nitrogen cycling [8].

The supplement of exogenous nitrogen is the necessary guarantee for plant production. Alfalfa is a leguminous plant that can directly absorb the ammonium nitrogen, nitrate nitrogen, and low organic nitrogen through symbiotic nitrogen fixation from soil [9]. However, the nitrogen fixed by nodules only accounts for 50% to 60% of its lifetime nitrogen demand, which cannot meet its growth needs [10]. Previous studies have demonstrated that alfalfa absorbs more ammonium ions than nitrate ions under hydroponic conditions, and obtains superior growth and root development with the increasing of ammonium nitrogen content [11,12]. In flooded and anaerobic soils, such as rice fields, the predominance of ammonium nitrogen has also been confirmed [13]. In contrast, alfalfa tended to absorb nitrate nitrogen under semi-arid and high-pH conditions where nitrification can occur to increase root and shoot biomass [14,15,16]. Therefore, alfalfa nitrogen uptake preference depends on soil environmental conditions (such as pH, soil water content, and inorganic nitrogen composition), which is closely related to the forms and proportion of exogenous nitrogen [17,18].

There are close links between the aboveground and belowground of soil; assays for plant characteristics, soil properties, and enzyme activities have become a common methodology for studying soil fertility and function in response to nitrogen fertilizer application. However, little is known about the response of aboveground and belowground to the different forms and proportions of exogenous nitrogen. Therefore, we performed a pot experiment of alfalfa to improve our understanding of how different forms and proportions of exogenous nitrogen affect the soil enzyme activities. We hypothesized that: (1) nitrogen forms and ratios significantly affect soil enzyme activities, (2) the fluctuation of enzyme activities leads to changes in soil nitrogen transformation, which, in turn, affects the growth of alfalfa. The verifications of the above hypotheses were expected to fill an important knowledge gap regarding the role of enzymes in plant–soil feedback and provide scientific guidelines for the rational use of nitrogen fertilizers, all of which will contribute to the sustainable development of agroecosystems.

## 2. Results

### 2.1. Soil Enzyme Activities

**Figure 1:**
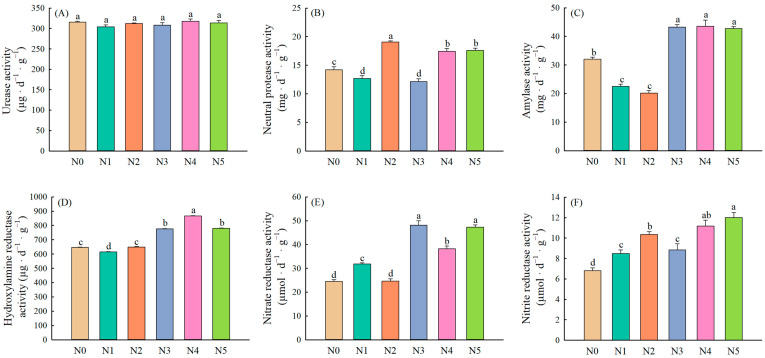
Soil enzyme activities under different forms and proportions of exogenous nitrogen ((**A**) Urease, (**B**) Neutral protease, (**C**) Amylase, (**D**) Hydroxylamine reductase, (**E**) Nitrate reductase, (**F**) Nitrite reductase). Values are means ± standard error (*n* = 4). Different letters indicate significant differences between different treatment groups at the 0.05 level. N0 (control), N1 (NaNO_3_), N2 (NH_4_Cl), N3 (NaNO_3_:NH_4_Cl = 7:3), N4 (NaNO_3_:NH_4_Cl = 1:1), N5 (NaNO_3_:NH_4_Cl = 3:7).

The results of specific variations of each enzyme under each treatment are shown in Figure 1. Neither single-nitrogen application nor mixed-nitrogen application significantly affected the activity of urease (Figure 1A). For the neutral protease, the positive effect of exogenous nitrogen on its activity was increased with the proportion of exogenous ammonium nitrogen, while single-nitrate nitrogen application had potential to depress enzyme activity (Figure 1B). It was found that mixed-nitrogen application markedly enhanced amylase activity, whereas the negative effect was observed in single-nitrogen application treatments (Figure 1C). Furthermore, hydroxylamine reductase and nitrate reductase activity in the soil increased significantly under exogenous nitrogen addition and the positive stimulation was more obvious in the mixed-nitrogen groups (Figure 1D,E). The nitrite reductase activity reached its highest value when the mixture of nitrate and ammonium nitrogen was 3:7 (Figure 1F).

**Figure 2:**
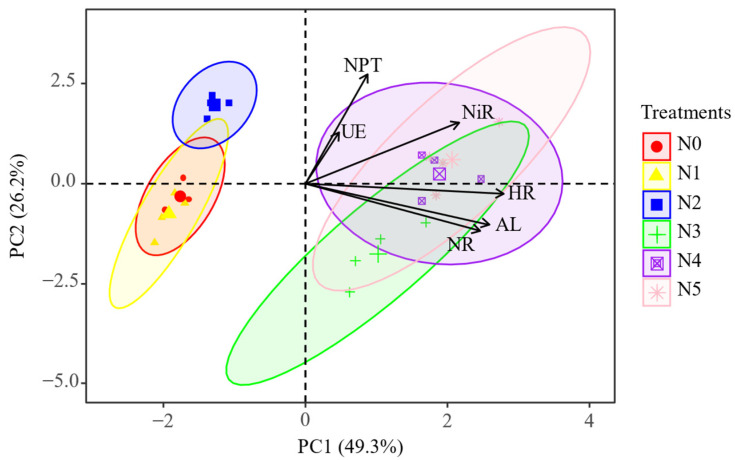
Principal component analysis of soil enzyme activities under different forms and proportions of exogenous nitrogen. N0 (control), N1 (NaNO_3_), N2 (NH_4_Cl), N3 (NaNO_3_:NH_4_Cl = 7:3), N4 (NaNO_3_:NH_4_Cl = 1:1), N5 (NaNO_3_:NH_4_Cl = 3:7). UE (urease), NR (nitrate reductase), NiR (nitrite reductase), NPT (neutral protease), AL (amylase), and HR (hydroxylamine reductase).

Different forms and proportions of exogenous nitrogen changed the enzyme activities in the soil. The effects of mixed-nitrogen addition and single-nitrogen addition on soil enzyme activities were markedly different. Mixed-nitrogen application significantly stimulated soil enzyme activities, whereas single-nitrate nitrogen application had no remarkable effect on the soil enzyme activities. Moreover, hydroxylamine reductase and amylase are two vital enzymes that cause significant overall differences in soil enzyme activities (Figure 2).

### 2.2. Soil Physicochemical Characteristics

**Figure 3:**
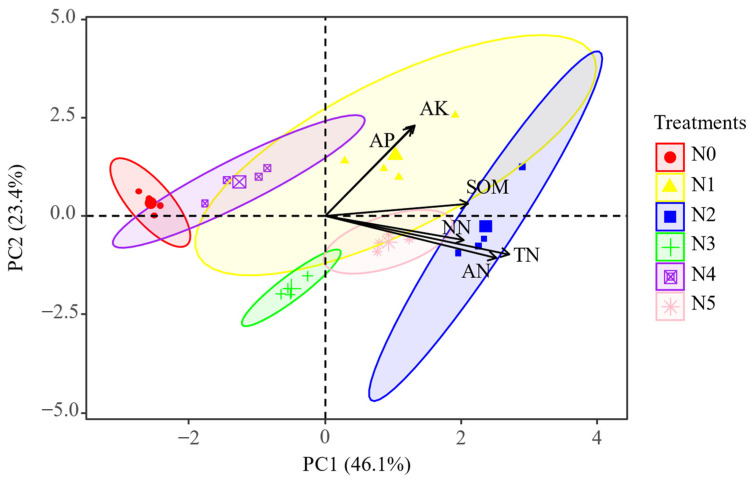
Principal component analysis of soil physicochemical properties under different forms and proportions of exogenous nitrogen. N0 (control), N1 (NaNO_3_), N2 (NH_4_Cl), N3 (NaNO_3_:NH_4_Cl = 7:3), N4 (NaNO_3_:NH_4_Cl = 1:1), N5 (NaNO_3_:NH_4_Cl = 3:7). SOM (soil organic matter), TN (total nitrogen), NN (NO_3_^−^-N), AN (NH_4_^+^-N), AP (available phosphorus), AK (available potassium).

The overall differences in soil physicochemical characteristics among six treatments were significant. Dim1 explained 46.1% of the variations, and the effect of TN was relatively large (Figure 3). The content of SOM was markedly higher in the single-nitrate nitrogen group than in others. Conversely, single-ammonium nitrogen application significantly increased TN content in comparison with other groups. SOM and TN reached the lowest value when the mixing ratio was 1:1. The content of soil NO_3_^−^-N increased by multiples under exogenous nitrogen addition from 5.84 to 16.16. It was found that soil NH_4_^+^-N value was highest under single-ammonium nitrogen addition, which was more than twice that of N0. Additionally, exogenous nitrogen significantly stimulated soil AP and AK (Table 1).

### 2.3. The Key Soil Factors Affecting Soil Enzyme Activities

**Figure 4:**
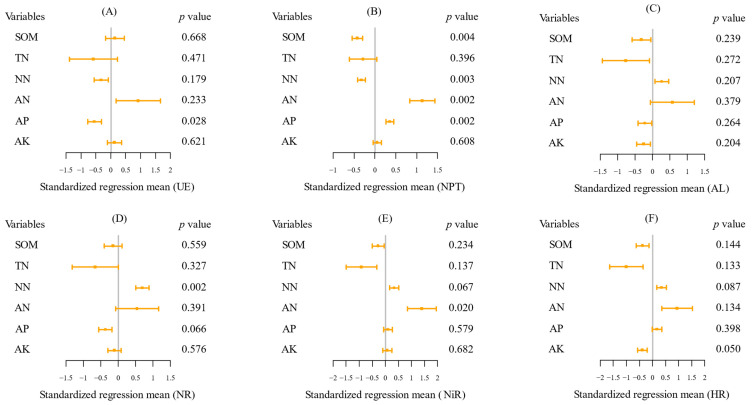
Results of multiple regression between soil enzyme activities and physicochemical properties ((**A**) Urease, (**B**) Neutral protease, (**C**) Amylase, (**D**) Nitrate reductase, (**E**) Nitrite reductase, (**F**) Hydroxylamine reductase). Each variable was standardized before comparing effect sizes (squares) to determine differences in the strength of predictor variables. Closed squares indicate mean of standardized regression coefficients and lines indicate standard errors. The abbreviations of variables are as follows: UE (urease), NR (nitrate reductase), NiR (nitrite reductase), NPT (neutral protease), AL (amylase) and HR (hydroxylamine reductase), SOM (soil organic matter), TN (total nitrogen), NN (NO_3_^−^-N), AN (NH_4_^+^-N), AP (available phosphorus), AK (available potassium).

Multiple regression results showed that soil characteristics contributed to the explained variation in enzyme activities (Figure 4). Based on the standardized regression coefficient and *p*-value, the activities of urease, nitrate reductase, nitrite reductase, and hydroxylamine reductase were significantly affected by soil AP (negative), NO_3_^−^-N (positive), NH_4_^+^-N (positive), and AK (negative), respectively (Figure 4A,D–F). NH_4_^+^-N (positive effect) explained the most variation in neutral protease activity (Figure 4B). Other variables with significant but small effects on neutral protease activity included SOM (negative), NO_3_^−^-N (negative), and AP (positive). NO_3_^−^-N and NH_4_^+^-N were the only two variables that promoted amylase activity, but the promotion effect was not significant (Figure 4C).

### 2.4. Plant Characteristics

**Figure 5:**
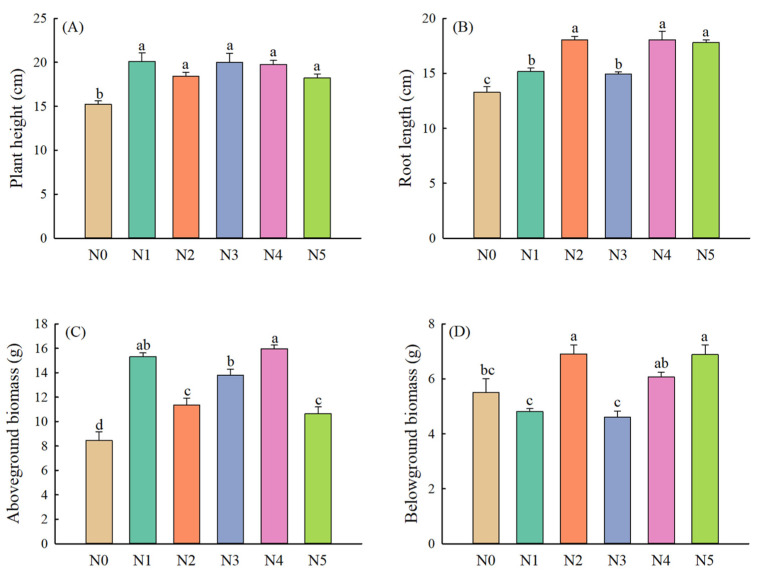
Growth and biomass differences of alfalfa under different forms and proportions of exogenous nitrogen ((**A**) Plant height, (**B**) Root length, (**C**) Aboveground biomass, (**D**) Belowground biomass). Values are means ± standard error (*n* = 4). Different letters indicate significant differences between different treatment groups at the 0.05 level. Note: N0 (control), N1 (NaNO_3_), N2 (NH_4_Cl), N3 (NaNO_3_:NH_4_Cl = 7:3), N4 (NaNO_3_:NH_4_Cl = 1:1), N5 (NaNO_3_:NH_4_Cl = 3:7).

**Figure 6:**
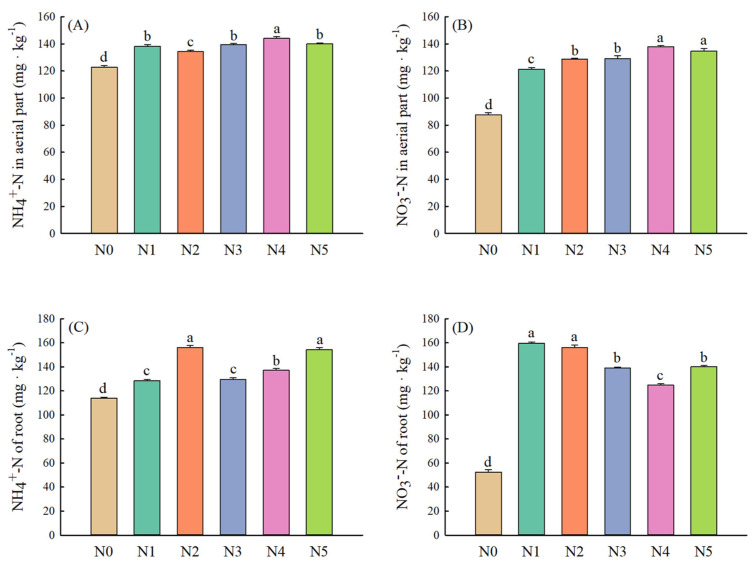
Nitrogen contents of alfalfa under different forms and proportions of exogenous nitrogen ((**A**) NH_4_^+^-N in aerial part, (**B**) NO_3_^−^-N in aerial part, (**C**) NH_4_^+^-N of root, (**D**) NO_3_^−^-N of root). Values are means ± standard error (*n* = 4). Different letters indicate significant differences between different treatment groups at the 0.05 level. Note: N0 (control), N1 (NaNO_3_), N2 (NH_4_Cl), N3 (NaNO_3_:NH_4_Cl = 7:3), N4 (NaNO_3_:NH_4_Cl = 1:1), N5 (NaNO_3_:NH_4_Cl = 3:7).

Compared with the control, different nitrogen application treatments significantly increased the plant height and root length of alfalfa (Figure 5A,B). The aboveground biomass increased significantly with the addition of exogenous nitrogen, while the negative effect of nitrogen application on belowground biomass was observed when nitrate nitrogen was applied alone and the ratio of mixing nitrogen was 7:3 (Figure 5C,D). Additionally, the content of NH_4_^+^-N and NO_3_^−^-N in the aerial part was highest when the mixing ratio was 1:1 (Figure 6A,B). Compared with other nitrogen application treatments, single-ammonium nitrogen application and single-nitrate nitrogen application exhibited significant positive effects on NH_4_^+^-N and NO_3_^−^-N contents of root, respectively (Figure 6C,D).

### 2.5. Relationships between Biomass of Alfalfa with Soil Properties and Enzyme Activities

**Figure 7:**
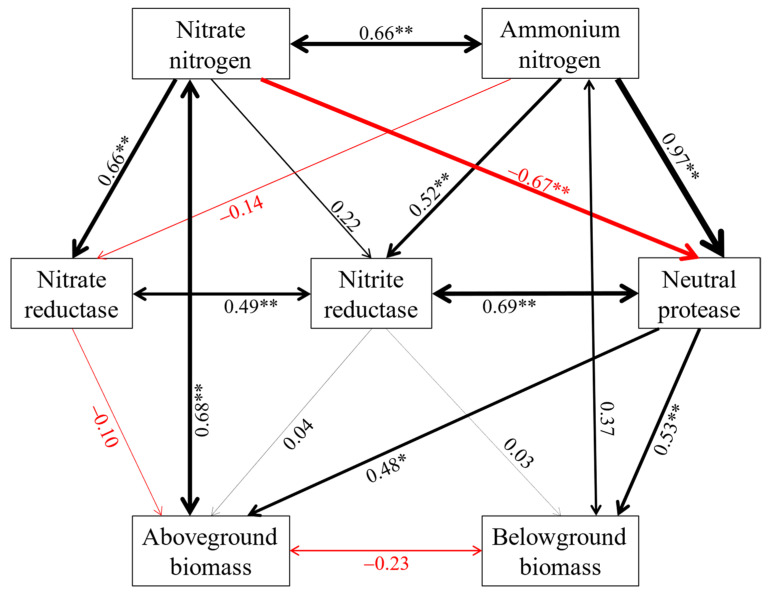
Structural equation model of direct and indirect effects of soil nitrate nitrogen and ammonium nitrogen enzyme activities on the biomass of alfalfa. The lines in black mean positive relationship, while those in red color mean negative relationship. The full and dashed lines represent direct and indirect effects, respectively. The width of lines is proportional to the strength of path coefficients. * and ** represent significant differences at the 0.05 and 0.01 level, respectively. This hypothetical model is consistent with our data, consistent with χ^2^ = 6.508, *p* = 0.164, GFI = 0.936, CFI = 0.979 values.

We used SEM analysis to explore the interactions between soil properties, enzyme activities, and aboveground biomass under different forms and proportions of exogenous nitrogen. As shown in Figure 7, the biomass of alfalfa directly and significantly altered soil nitrogen content. There existed one primary obvious path leading to the increase in aboveground biomass and belowground biomass, respectively. The addition of exogenous nitrogen significantly stimulated the nitrite reductase activity by increasing the soil nitrogen content, thereby enhancing the aboveground biomass of alfalfa. Meanwhile, soil NH_4_^+^-N significantly increased the activity of neutral protease, which accelerated the turnover of nitrogen in the soil and ultimately increased belowground biomass. However, soil NO_3_^−^-N showed a direct and significant negative effect on neutral protease activity.

## 3. Discussion

The specific transformation process and mechanism of nitrogen fertilizer in the plant–soil system are regulated by many factors. As important biological activators of underground energy flow and material circulation, soil enzymes participate in the conversion of soil nutrients [19,20]. Changes in soil enzyme activities can well reflect the changes in soil physicochemical properties and energy metabolism, which can then affect the growth of plants [21,22,23].

### 3.1. Effects of Different Forms and Proportions of Exogenous Nitrogen on Hydrolase Activity

Urease is a hydrolase that has a crucial effect on the soil nitrogen cycle. To be more specific, it transforms small nitrogen-containing organic substrates into inorganic nitrogen-containing compounds (such as ammonia) to provide available nitrogen for the normal growth and development of plants [24]. Several studies had documented that nitrogen addition often enhanced urease activity [23,25]. Enhancement of urease activity under nitrogen addition may be due to the increase in soil carbon and nitrogen availability that stimulates microbial activity, as well as a more suitable soil environment for microbial growth and proliferation that benefits from the improvement of soil physicochemical properties [26,27]. However, negative effects had also been observed [28], which was a consequence of lower pH [29] caused by long-term nitrogen fertilizer. In our study, neither single-nitrogen application nor mixed-nitrogen application significantly affected urease activity (Figure 1A), which was consistent with the result of a previous nitrogen fertilization study [30]. A possible explanation for this is that a high concentration of NH_4_^+^ that comes from the nitrogen fixation of alfalfa hinders the synthesis of urease [31].

Neutral protease participates in the turnover of nitrogen in the soil by hydrolyzing proteins into oligopeptides and amino acids, which is considered to be the rate-limiting process of soil nitrogen mineralization [32]. Our results showed that the treatment of exogenous nitrogen had both significant positive and negative effects on the activity of soil neutral protease (Figure 1B). In particular, the protease activity significantly increased when NH_4_^+^ was the preferred N source. However, another study showed that NH_4_^+^ had a significant negative effect on the activity of protease [33]. The inconsistent response of neutral protease activity to the addition of exogenous nitrogen might be related to the nitrogen uptake preference of alfalfa. Ammonium nitrogen and nitrate nitrogen are present in soil solution as NH_4_^+^ and NO_3_^−^ until they are absorbed and utilized by alfalfa [14]. Studies [34,35] have shown that roots release an equivalent amount of H^+^ in exchange for NH_4_^+^ when plants assimilate ammonium nitrogen. In comparison, NO_3_^−^ repel each other with negative charges in the soil, which contributes to the leaching loss of nitrate nitrogen [36]. Meanwhile, evidence [37] suggests that energy consumption can differ in the assimilation of NH_4_^+^ (5 ATP mol^−1^) and NO_3_^−^ (20 ATP mol^−1^). Thus, the activity of neutral protease increased in N2, N4, and N5 but decreased in N1 and N3. Furthermore, plant nitrogen uptake preference indirectly influences the availability of carbon and nitrogen through effects on root exudates and microbial biomass [38]. For instance, application of nitrogen fertilizer alleviated the nitrogen limitation of the Korean pine forests [39] and there was no need for microorganisms to release nitrogen through decomposing organic matter by mineralization so that the neutral protease activity was significantly reduced. On the contrary, the efficient utilization of nitrogen not only enhanced mineralization, but also increased the release of root exudates, which together stimulated the activity of neutral protease [40,41]. The exogenous nitrogen changes the distribution pattern of soil carbon and nitrogen, while proteases decompose proteins from plant litter, root exudates, and microbial residues to maintain the availability of soil carbon and nitrogen [38,41,42]. This reveals the crucial role of microbe-mediated protein mineralization in soil carbon and nitrogen coupling cycles, and NH_4_^+^ as the final product of mineralization indirectly reflects protease activity [38].

Amylase is the main catalyst for the decomposition of soil organic matter and humus, which plays a primary role in soil carbon and nitrogen cycle [43]. The activity of amylase is influenced by soil fertility, which is closely related to vegetation types and soil management measures. The previous reviews [44] found that vegetation degradation had a negative impact on amylase activity, whereas natural and improved fallows significantly increased it [45]. Fertilization is one of the most basic approaches to improve soil fertility, and Samal [46] revealed that low-concentration urea treatment was helpful in increasing amylase activity, while high-concentration treatment inhibited it. The type and proportion of nitrogen fertilizer also influenced the response of enzyme activities to fertilization. Jaspreet Kaur et al. [47] found that mixed-nitrogen fertilizer addition exerted a significantly positive influence on amylase activity compared to single-nitrogen fertilizer application, which was consistent with our research results (Figure 2C). However, in the previous study, the application of nitrogen fertilizer regardless of the form and proportion significantly improved soil amylase activity [48]. Both single application and combined application input exogenous organic matter to the soil directly to increase the content of soil organic carbon. The accumulation of organic matter and the spatial heterogeneity of soil substrate caused by the stratification of organic matter provides a large amount of carbon and nitrogen substrates and foraging niches for the survival of soil microorganisms, respectively [49,50]. As for the inhibitory effect of single-nitrogen application on amylase activity, a convincing explanation is the competition between plants and microorganisms for nitrogen. Cui [51] et al. observed that long-term application of fertilizers decreased soil nitrogen availability with increasing accumulation of soil carbon. Based on this situation, microorganisms and plants are simultaneously exposed to soil nitrogen limitation and will compete for nitrogen; soil C/N ratio consequently increased. The enhancement of C/N ratio caused by nitrogen competition inhibits the release of amylase by microorganisms, while the decrease in litter quality makes the soil organic matter quite recalcitrant, which depresses the activity of amylase [25,42,51,52].

### 3.2. Effects of Different Forms and Proportions of Exogenous Nitrogen on Oxidordeuctase Activity

Nitrate reductase and nitrite reductase are the regulating enzymes and rate-limiting enzymes of nitrate assimilation in plants, which are not only sensitive to external nitrogen, but also indirectly make a difference to the uptake and utilization of nitrogen in plants [53]. Specifically, nitrate reductase and nitrite reductase catalyze NO_3_^−^ and NO_2_^−^ and transform them into NO_2_^−^ and NO, respectively [54]. Nitrite will form intermediate product hydroxylamine in the process of being reduced, and hydroxylamine is reduced to ammonia under the action of hydroxylamine reductase [54,55]. The mineralization, nitrification, and denitrification induced by these enzymes are the key biochemical processes that affect the nitrogen utilization, loss, and greenhouse gas emissions in the process of soil nitrogen metabolism [56,57]. Therefore, various factors that affect soil nitrogen metabolism also have been considered as the major factors determining the activity of these enzymes, especially fertilization. Fertilization can directly influence soil properties and soil microbial composition and abundance, and further indirectly affect soil enzyme activities. Wang et al. [57] reported the promotion of nitrate reductase activity in a paddy ecosystem by elevated nitrogen fertilizer level, but the mixed application of pig manure and nitrogen fertilizer significantly inhibited the activities of nitrate reductase and nitrite reductase, while it increased hydroxylamine reductase activity [53]. This may be due to the inhibitory effect of high C/N ratio on the growth of soil denitrifying bacteria, which results in the accumulation of hydroxylamine [58]. In our experiment, activities of these three enzymes increased in each treatment and were higher than that in control, and the enzyme activities were positively associated with the soil nitrate nitrogen, which was the substrate of redox reaction (Figure 1D–F and Figure 3). This was in line with the results of Weng et al. [23]. Furthermore, it was also found in our experiment that the positive effects of mixed-nitrogen application on enzyme activities were better than those of single-nitrogen application (Figure 1D–F). We might attribute the phenomenon to the difference in the type and quality of rhizosphere exudates, which related to nitrogen forms and proportions. Previous research revealed that the root exudates induced by alfalfa root growth caused a positive priming effect, and then increased the soil microbial population and nitrogen requirement [59], which was a plausible explanation for the higher activities of nitrate reductase, nitrite reductase, and hydroxylamine reductase.

The results of our study showed that there were strong interactions between biomass of alfalfa and soil available nitrogen content, while nitrate reductase, nitrite reductase, and neutral protease were the three vital acting enzymes (Figure 7). This is because alfalfa influences the transformation of soil organic matter through root growth, root exudates, and litter. In turn, as the main drivers of the organic matter decomposition process, enzymes have a direct impact on soil fertility, thereby affecting the absorption efficiency of soil nutrients by alfalfa. In addition, it was interesting to note that, when a 1:1 ratio of nitrate to ammonium nitrogen was applied, soil nutrient content and enzyme activities were lower than the other treatments, but the plant tissue nutrient content and biomass were comparable to others (Figure 1, Figure 5, and Figure 6; Table 1). A possible explanation for this was that alfalfa was in its first flowering period at the time of sampling in this experiment and soil nutrients were always preferentially available for aboveground growth and development [34]. These empirical findings are a good reflection of the “plant-soil feedback” theory.

## 4. Material and Methods

### 4.1. Experiment Design and Soil Sample

The pot experiments were carried out in the greenhouse of the Pratacultural Science Department of Yangzhou University, Jiangsu Province, China (119°43′ E, 32°39′ N). The region has a mild climate all year round and belongs to the subtropical humid climate zone. The average annual temperature of the city is 14.7–15.5 °C and the total annual precipitation is 950–1026 mm [60]. The alfalfa variety was WL233 (Zhengdao Seed Industry Co., Ltd., Life Garden Road, Huilongguan Town, Changping District, Beijing) and the specification of the pot was 18 × 16 × 24 cm. Ten alfalfa seeds were evenly spotted in each pot. The soil (sandy loam soil type) for the pots was selected from 0 to 30 cm deep in the field outside the greenhouse of the Pratacultural Science Department of Yangzhou University. Basic agrochemical characteristics of soil in the pots were as follows: soil organic matter, 11.15 g·kg^−1^; ammonium nitrogen, 4.85 mg·kg^−1^; nitrate nitrogen, 8.68 mg·kg^−1^; total nitrogen, 0.76 g·kg^−1^; available phosphorus, 35.42 mg·kg^−1^; available potassium, 72.53 mg·kg^−1^; pH 8.13.

Different forms and proportions of nitrogen were treated by regulating the nutrient solution: N0 (control, nitrogen-free nutrient solution), N1 (NaNO_3_), N2 (NH_4_Cl), N3 (NaNO_3_:NH_4_Cl = 7:3), N4 (NaNO_3_:NH_4_Cl = 1:1), N5 (NaNO_3_:NH_4_Cl = 3:7). Each treatment had 4 replicates and a total of 24 pots. The contents of nitrogen-free nutrient solution were MgSO_4_·7H_2_O 0.12 g, KH_2_PO_4_ 0.1 g, Na_2_HPO_4_·12 H_2_O 0.15 g, EDTA-Fe 0.005 g, and CaCl_2_·2H_2_O 0.1 g; then, 1 mL of Gibson trace element liquid was added and diluted with distilled water to 1000 mL, which adjusted the pH to 7.0. The pure nitrogen concentration of the nutrient solution in each pot was 210 mg.L^−1^.

Irrigation, weed control, and other management measures of each pot were the same to ensure the normal growth of alfalfa. The nutrient solution was applied at the three-leaf stage, once a week and 200 mL each time, for a period of six weeks. Aboveground biomass was determined by clipping all plants within a pot and weighing the fresh weight at budding stage. Belowground biomass was determined by washing with distilled water, drying with absorbent paper, and weighing. The content of ammonium nitrogen and nitrate nitrogen in plant roots and leaves were determined using a kit (Suzhou Keming Biological Co., Ltd., No.218, Xinghu Street, Suzhou Industrial Park, Jiangsu, Suzhou, China). After surface impurities were removed, the soil was collected from each pot using a soil auger (4 cm diameter, 20 cm depth). Then, each of the soil samples was passed through a 2-mm sieve and divided into two parts. One of them was air-dried to determine soil organic matter, total nitrogen, available phosphorus, and potassium. The other part was stored at 4 °C immediately for analyzing soil enzyme activities and soil ammonium and nitrate nitrogen values.

### 4.2. Soil Properties

The determination of soil physicochemical properties refers to “Soil Sampling and Methods of Analysis” [61]. Soil organic matter (SOM) was measured by using the K_2_Cr_2_O_7_ redox titration method. Total nitrogen (TN) in the soil was determined by Kjeldahl digestion. Soil ammonium nitrogen (NH_4_^+^-N) was determined by the indophenol blue colorimetric method. Nitrate nitrogen (NO_3_^−^-N) in the soil was determined by the phenol disulfonic acid colorimetric method. Olsen method and flame photometric method were used to extract available phosphorus (AP) and available potassium (AK), respectively.

### 4.3. Soil Enzyme Activities

Urease (UR) activity was assessed by incubating a 5 g fresh soil sample for 2 h at 37 °C with 2.5 mL of a 0.08 M urea solution, and NH_4_^+^ was later determined by spectrophotometer at 690 nm [62]. The urease activity was expressed as μg NH_4_^+^-N d^−1^ g^−1^. The amylase (AL) activity and neutral protease (NPT) activity were analyzed using the 3,5-dinitrosalicylic acid colorimetric method and the ninhydrin colorimetric method, respectively [63,64]. The amylase activity and neutral protease activity were expressed as mg sugar reduced d^−1^ g^−1^ and mg tyrosine produced d^−1^ g^−1^, respectively. Nitrate reductase (NR) activity was measured according to Abdelmagid [65] using KNO_3_ as the substrate, and the value of it was calculated with the content of NO_3−_ reduction after 24-h incubation at 25 °C. The nitrite reductase (NiR) activity was determined using NaNO_2_ as a substrate, and its activity was expressed as decreasing μmol NO_2−_ d^−1^ g^−1^ after a 24 h anaerobic incubation of soil samples at 30 °C [23]. The phenanthroline ferrous sulfate method was used to determine hydroxylamine reductase activity, and the activity was expressed as μg hydroxylamine d^−1^ g^−1^ [23].

### 4.4. Statistical Analysis

The data were analyzed with statistical methods as follows: (1.) The differences in soil enzyme activities, soil properties, and alfalfa characteristics were tested using one-way analysis of variance (ANOVA), followed by Duncan’s new multiple range. These analyses were performed with the IBM Statistical Package, SPSS version 25.0 (IBM, Armonk, NY, USA), and then histogram plots were plotted by SigmaPlot software. (2.) Principal component analysis was used to analyze the overall differences in soil properties and enzyme activities under different nitrogen treatments. (3.) Multiple regressions were used to further analyze responses of enzyme activities to changes in soil physicochemical properties. (4.) A structural equation model was constructed to explore the relationships between enzyme activities, soil properties, and biomass of alfalfa. The above analyses (2–4) were conducted with R software, version 4.1.0.

## 5. Conclusions

In this study, we found that nitrogen forms and proportions significantly affected the activities of soil enzymes, except for urease. In particular, single-ammonium-nitrogen application significantly improved the neutral protease activity, while the mixed-nitrogen application had a remarkable positive stimulation on the activities of amylase, nitrate reductase, nitrite reductase, and hydroxylamine reductase. The results showed that application of different forms and proportions of nitrogen accelerated the turnover rate of soil nitrogen through enhancing the soil enzyme activities. The content of soil nutrients, mainly including soil organic matter, total nitrogen, etc., were significantly higher than the control group, and the increased enzyme activities and soil nutrient availability jointly promoted the growth of alfalfa. In summary, nutrient cycling in an ecosystem is a series of complex processes, and soil enzymes are involved in most of the processes, which link the quality of soil nutrients, the ability of microbes to use carbon for self-metabolism, and plant health.

## Figures and Tables

**Table 1:** Effects of different forms and proportions of exogenous nitrogen on soil physicochemical characteristics.

Items	Treatments					
	N0	N1	N2	N3	N4	N5
Organic matter (g kg^−1)^	13.80 ± 0.11 ^bc^	15.58 ± 0.65 ^a^	15.25 ± 0.22 ^a^	14.80 ± 0.15 ^ab^	13.29 ± 0.30 ^c^	14.15 ± 0.15 ^bc^
Total N (g kg^−1^)	0.73 ± 0.01 ^d^	0.80 ± 0.01 ^c^	0.93 ± 0.00 ^a^	0.80 ± 0.01 ^c^	0.74 ± 0.01 ^d^	0.87 ± 0.01 ^b^
NO_3−_N (mg kg^−1^)	5.84 ± 0.08 ^d^	16.16 ± 0.51 ^a^	13.81 ± 0.71 ^b^	15.64 ± 0.44 ^a^	11.95 ± 0.63 ^c^	13.88 ± 0.52 ^b^
NH_4+_-N (mg kg^−1^)	2.09 ± 0.02 ^e^	2.83 ± 0.02 ^d^	5.19 ± 0.04 ^a^	3.15 ± 0.01 ^c^	2.76 ± 0.01 ^d^	4.57 ± 0.05 ^b^
AP (mg kg^−1^)	40.65 ± 0.43 ^b^	44.61 ± 1.30 ^a^	44.72 ± 0.56 ^a^	39.41 ± 0.39 ^b^	45.02 ± 0.91 ^a^	41.13 ± 0.49 ^b^
AK (mg kg^−1^)	69.88 ± 0.13 ^c^	78.25 ± 0.43 ^a^	72.03 ± 2.52 ^bc^	62.54 ± 0.21 ^d^	70.20 ± 0.53 ^bc^	73.56 ± 0.66 ^b^

Note: values are means ± standard error (*n* = 4). The superscript letters “a, b, c, d, e” represent statistically significant differences (*p* < 0.05). N0 (control), N1 (NaNO_3_), N2 (NH_4_Cl), N3 (NaNO_3_:NH_4_Cl = 7:3), N4 (NaNO_3_:NH_4_Cl = 1:1), N5 (NaNO_3_:NH_4_Cl = 3:7).

## Data Availability

The data presented in this study are available on request from the corresponding author (snsun@yzu.edu.cn). The data are not publicly available due to the data presented in this study are also used in other unpublished paper.
